# As Radical as Technically Feasible—Surgical Treatment for Mobile Spine Chordoma

**DOI:** 10.3390/cancers17121989

**Published:** 2025-06-14

**Authors:** Alexander Lars Quiring, Hraq Sarkis, Paul Backhaus, Maximilian von Oldershausen, Bernhard Meyer, Nicole Lange

**Affiliations:** Department of Neurosurgery, Klinikum Rechts der Isar, Technical University of Munich, 81675 Munich, Germanybernhard.meyer@tum.de (B.M.)

**Keywords:** chordoma, spine, en bloc resection, intralesional surgery, radiotherapy

## Abstract

**Purpose:** In this retrospective study, we compared the impact of en bloc resection with negative margins to that of intralesional resection followed by adjuvant radiotherapy on local control (LC) and overall survival (OS) in patients with mobile spine chordomas. Secondary endpoints included mechanical complication rates, associated risk factors, and quality of life outcomes. **Methods**: Between June 2008 and March 2025, 26 patients aged ≥ 15 years with mobile spine (C1–L5) chordomas underwent surgical treatment at our institution. Patients were divided into en bloc resection (=Enneking appropriate (EA)) and intralesional resection (=Enneking inappropriate (EI)) plus HT groups. Clinical, oncologic, and surgical data were collected and analyzed to determine outcomes. **Results**: The EA group demonstrated a clear trend toward improved local recurrence-free survival (LRFS, EA median 35 months vs. EI resection median 14 months) and OS. Adjuvant therapy led to better LRFS and OS independently of the extent of resection. Clinical outcomes and quality of life were favorable in both groups, with no statistically significant differences in short-term, as well as long-term complications (27%). No specific risk factors for long-term complications were identified. **Conclusions**: En bloc resection remains the gold standard for mobile spine chordoma resection whenever feasible. Nevertheless, Enneking-appropriate resection is often limited by anatomical constraints. In such cases, every effort should still be made to achieve the most complete tumor resection possible prior to initiating adjuvant therapy. Due to the rarity of spinal chordomas and their surgical complexity, these procedures should be performed in specialized spine centers with multidisciplinary expertise.

## Simple Summary

Mobile spine chordomas are rare, slow-growing bone tumors that can be challenging to treat due to their location and the risk of spreading to nearby structures. This study aimed to compare two main surgical approaches for removing these tumors: en bloc resection, which aims to remove the tumor in one piece with negative margins, and intralesional resection, which involves removing the tumor in smaller parts but often leaves some tumor cells behind. The goal was to understand which method leads to better patient outcomes, including survival and quality of life, while also evaluating potential complications. The findings suggest that complete en bloc resection, when possible, provides the best chance for long-term control of the disease, although it is not always feasible due to the tumor’s location. This research supports the importance of highly specialized centers for the treatment of these complex cases.

## 1. Introduction

Chordomas are rare, low-grade notochordal tumors with a predilection for the axial skeleton. Due to indolent growth patterns, the encapsulation of adjacent neurovascular structures, and extensive tumor cell dissemination within the resection cavities, they behave malignantly, posing challenges in treatment strategies [1]. The involvement of the mobile spine (C1–L5) is uncommon, accounting for roughly 20% of all cases [2,3]. Surgical resection, performed as completely as technically feasible, remains the cornerstone of treatment. En bloc resection with negative margins (Enneking appropriate (EA)) has been shown to offer the most favorable oncologic outcomes, representing the suggested treatment for virgin and recurrent lesions [4].

However, due to their inherent invasiveness, en bloc resections show high complication rates, leading to more restrictive and patient-tailored decision making and urging surgical treatment in specialized spine centers. Particularly in the mobile spine, it is often limited by anatomical constraints, which frequently precludes the achievement of wide surgical margins.

Moreover, evidence underlining the critical role of charged-particle therapy in a variety of indications in chordoma therapy is on the rise. It has significantly improved local control (LC) in otherwise unresectable or incompletely resected tumors and has therefore emerged as an essential adjuvant therapy. These factors have also contributed to a growing debate in recent years questioning the necessity of aggressive surgical resection [5].

In this study, we present the oncological and functional outcomes of a large modern patient cohort that underwent surgery for chordomas of the mobile spine, based on a retrospective analysis, and highlight the feasibility as well as the significance of surgical resection followed by adjuvant therapy.

## 2. Materials and Methods

In this study, we conducted a retrospective analysis of 26 consecutive patients who were treated for chordomas of the mobile spine (C1–L5) between May of 2009 and March of 2025. Exclusion criteria were more than one previous surgical treatment and age <15 years. Of these patients, 8 were treated in our facility for recurrence of a known chordoma, and the other 18 presented with virgin tumors. A multidisciplinary tumor board (including the heads of the departments of neurosurgery, neuroradiology, oncology, and radiotherapy, as well as all responsible consultants) reviewed all cases, and all newly diagnosed tumors (solitary tumors with no prior known cancer) underwent a CT-guided biopsy to confirm the diagnosis. Based on considerations of metastases, prognosis, comorbidities, and the extent of lesion, the decision for feasibility of en bloc resection or necessity of intralesional surgery was made [4,6,7]. In 2 cases of patients with acute neurological deficits, tumor debulking was performed as an emergency surgery. After histopathologic diagnosis of chordoma had been confirmed, the tumor board decided on complete resection and adjuvant radiotherapy.

En bloc resections were performed as follows (as described in: *Two-stage en bloc vertebrectomy for primary spinal bone tumors: A surgical strategy for optimal resection with low complication rates*) [8].

In the cervical spine, monosegmental iCT-navigated dorsal instrumentation with pedicle screws (Neon-Ulrich medical, Ulm, Germany) was conducted, followed by a dorsal release (laminectomy, facetectomy, and skeletonization of vertebral artery). In patients with a tumor surrounding the vertebral artery, endovascular closure was performed before surgery. The second step comprised en bloc resection and stabilization with a PEEK implant (Athlet-Ulrich medical, Ulm, Germany) or fibula bone graft via an anterior approach.

Patients with lesions of the thoracical and lumbar spine were first operated on via a posterior approach in a prone position. A navigation-guided (Brainlab neuronavigation, Brainlab AG, Munich, Germany), percutaneous insertion of pedicle screws was performed two levels above and below the affected vertebra. Afterward, a laminectomy was performed, along with the resection of the transverse process and pediculectomy in the lumbar spine or costotransversectomy as well as pediculectomy in the thoracic spine. Segmental arteries were clipped, and a blunt dissection toward the anterior border was carried out from both sides. Gauze was circumferentially placed around the affected vertebrae on both the ipsilateral and contralateral sides and left in situ to separate layers and aid in orientation for the subsequent en bloc resection. Finally, stabilization was completed by placing the rods.

The second step was performed within a few days. The patients were positioned laterally at a 90-degree angle. A retroperitoneal or transthoracic approach to the spine was performed, and with the guidance of iCT and navigation, the affected vertebra was exposed and the initial left behind gauze was removed. Intervertebral discs were incised and completely removed, and the anterior border was bluntly prepared until the contralateral gauze was reached. The vertebral body was then removed en bloc, and a carbon fiber expandable cage (Kong-TL VBR, Icotec, Altstaetten, Switzerland) was implanted. ICT was performed to confirm the resection and the implant’s accurate positioning.

Intralesional resections were emergency procedures due to acute neurological deficits in two cases. They received dorsal stabilizations and tumor debulking via laminectomy within 12 h. In the remaining patients, opening of the tumor capsule or incomplete resection was done due to an anatomic situation or to minimize invasiveness.

All lesions were characterized radiographically using the SINS score [9], the Enneking classification system for benign and malignant tumors [2], and the Weinstein–Boriani–Biagini classification [10]. According to the Enneking classification system, tumors were classified based on their grade (1: low versus 2: high) and local extension (A: intra- vs. B: extra-compartmental), as well as 3 for systemic metastases [11,12]. The largest diameter of the tumor in the sagittal MRI was recorded.

We classified en bloc resections as Enneking appropriate (EA) and R0 and intralesional resections as Enneking inappropriate (EI). Cases of macroscopically completely removed tumors are referred to as R1 and cases of visual tumor remnants as R2 [13].

The duration of surgery (first, second, and total), blood loss, mortality, complication rates, length of hospital stay (LOH), overall survival (OS), and local recurrence-free survival (LRFS) were assessed. The Karnofsky performance status scale (KPS) score at admission and during the follow-up (after 3 and 6 months and again 1, 3, and 5 years after surgery, depending on the initial surgery date) was used to account for outcomes.

Statistical analyses, including descriptive data analyses, were conducted using IBM SPSS Statistics version 29.0 (IBM Corporation, New York, NY, USA). Descriptive statistics for demographic variables were generated with means and SDs or medians with interquartile ranges, as appropriate.

Survival time was calculated from the primary surgery to death or the last contact date. Time to local recurrence was calculated from primary surgery to the first tumor recurrence or the last contact date. All survival data was described with Kaplan–Meier curves.

The local Ethics Committee approved this study, which was conducted according to the ethical standards established by the 1964 Declaration of Helsinki and its later amendments [14] (clinical trial registration number 205/18S). This study was a retrospective analysis, so the Ethics Committee waived patients’ informed consent.

## 3. Results

The results in the table below are comprehensively summarized, comparing the EA (Enneking appropriate) and EI (Enneking inappropriate) groups, with consideration of the corresponding statistical *p*-values.

### 3.1. Clinical Characteristics

Between 2009 and 2025, 26 patients were treated for chordoma of the mobile spine at our institution. The cohort had a mean age of 57 years (ranging from 16 to 88), and 19 patients were male (73.1%). The mean BMI was 28.41 in the EA group and 26.97 in the EI group (*p* = 0.93), and the mean Charlson Comorbidity Index score was 4.63 in the EA group and 5.07 in the EI group (*p* = 0.71). The clinical characteristics of the two cohorts are shown in Table 1 and showed no significant differences between groups.

Radiographically, the mean SINS score was nine (ranging from 6 to 14). Based on the Enneking classification system, one tumor was classified as 1A (4%), four lesions as 1B (15%), two lesions as 2A (8%), and fourteen lesions as 2B (54%). In five patients (19%), staging showed systemic metastases (Enneking 3).

The mean tumor size, assessed via sagittal T2 MRI sequences as the largest diameter, was 40 mm (ranging from 18 to 94 mm). Notably, 73% of the patients showed extraosseous tumor growth with intraspinal involvement, characterized as Weinstein–Boriani–Biagini D or E. Tumor location was cervical in thirteen cases (50%), thoracic in three cases (11.5%), and lumbar in ten cases (38.5%).

Clinical presentation was due to back pain in 84% of the patients, accompanied by sensory deficits in seven cases. Two patients presented with severe neurological symptoms (ASIA B) that made timely decompression of the spinal canal as part of emergency surgeries necessary.

A total of eighteen patients presented with primary chordoma, while eight exhibited recurrences of previously diagnosed chordomas. These patients had undergone prior intralesional resections at outside institutions, ultimately precluding the possibility of an en bloc resection. The five patients with metastases at the time of presentation were all resected EI.

### 3.2. Treatment

**Figure 1:**
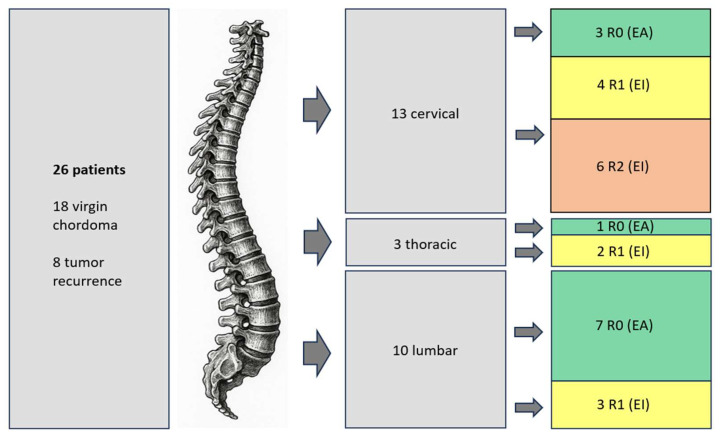
Treatment strategy of patients. R0: Enneking-appropriate resection with wide surgical margins; R1: Enneking-inappropriate resection with microscopically positive margin; R2: EI resection with macroscopically positive margin.

The treatment strategy of the cohort is illustrated in Figure 1. Table 2 shows a summary of all cases, their surgical strategy and outcomes, as well as the follow-up.

After discussion of all cases in our interdisciplinary tumor board, the cohort was treated as follows:

With regard to cervical chordomas, en bloc resection was achieved in three cases. In an additional four patients, no macroscopic tumor remnants were detected; however, intraoperative violation of the tumor capsule occurred, resulting in R1 resections. The remaining six patients demonstrated macroscopic residual tumor (R2). These cases were as follows:

1. A young patient with an incidental finding of the tumor during routine imaging follow-up for familial cavernomatosis. Given the patient’s pre-existing condition and the minimal residual tumor burden on imaging, the multidisciplinary tumor board recommended carbon ion therapy in lieu of further surgery.
2. A patient with a complicated postoperative course, including fulminant ventriculitis, following prior surgery abroad. Due to the clinical condition, re-resection was deemed unfeasible.
3. Two patients had undergone prior surgery abroad and presented with distant metastases at the time of evaluation, precluding further aggressive surgical intervention.
4. Postoperative imaging revealed residual bony tumor fragments, which were completely resected during a third surgical procedure.
5. A preoperative balloon occlusion test yielded negative results, indicating that occlusion of the vertebral artery was not tolerated, thus rendering an en bloc resection technically impossible.

All three patients with thoracic lesions underwent complete resection, with one achieving R0 status and two classified as R1 resections as the tumor capsule was in contact with the thoracic aorta in one case, and as emergent decompression surgery had already taken place in another patient, so that complete resection was achieved after histopathological proven chordoma.

Of the 10 patients with lumbar chordomas, seven underwent en bloc resection. In the remaining three patients, although gross total resection was achieved, intraoperative violation of the tumor capsule occurred, resulting in R1 resections. (One received open biopsy in another hospital, one received partial tumor resection in another hospital, one patient underwent surgery under the presumptive diagnosis of a hemangioma, and definitive complete resection was only performed secondarily after histopathological confirmation of the chordoma).

Mean cumulative blood loss for both surgical steps was 3008 mL (ranging from 180 to 15,000 mL), and mean procedure duration was 378 min (ranging from 73 to 912 min). As expected, blood loss in the EA group was significantly higher (4690 mL) than in the EI group (1775 mL), *p* = 0.027. Mean procedure duration did not differ significantly (468 min in the EA group vs. 312 min in the EI group), *p* = 0.078. There were no major intraoperative complications.

Investigation of the complications during the hospital stay showed no significant differences between the groups. There were two wound healing disorders (both from the EI group) that required revision under local anesthesia, one case of pulmonary embolism, which was successfully treated with heparin, and one case of meningitis with ventriculitis (one of EI and one of EA group). In one case, postoperative CT imaging showed malpositioning of the carbon cage, which had to be repositioned (short-term complication rate of 19%). Mean LOH was 21 days in both groups (range 5 to 60 days, *p* = 0.9).

A total of 17 patients underwent adjuvant radiotherapy after consensus in our interdisciplinary tumor board (of the remaining nine patients, six had already received radiotherapy due to their first chordoma therapy; two went back home abroad with the recommendation to receive charged-particle therapy and one patient refused to receive radiotherapy and decided for MRI control—he experienced a tumor recurrence 161 days after surgery). Interestingly, patients in the en bloc resection group showed a trend to initiate radiotherapy more rapidly, thus without statistical significance (mean time to therapy: 28 days, vs. 40 days in the EI group), *p* = 0.49.

Concerning neurological outcomes, all nine patients with previous sensory or motor deficits improved (six of them were in the EA group, three were in the EI group). Back pain improved in 13 patients. One patient declined (patient ID 24—he developed severe meningitis and was treated with intravenous antibiotics with meropenem and vancomycin. He was subsequently transported back home abroad with a final KPS of 60 after a few weeks in our ICU. There he achieved good recovery and was treated with radiotherapy. The follow-up ends after 358 days without tumor progression).

The mean quality of life measured via KPS was 80 (ranging from 50 to 90) in the EA group and 90 (ranging from 40 to 100) in the EI group before surgical treatment. No significant difference was observed (*p* = 0.34). Quality of life remained at 80 (50–90) in the EA group at the time of release. In the EI group, it decreased to 70 (0–100). There were no significant changes in KPS over the course of the surgical treatment (EA: *p* = 0.82; EI: *p* = 0.42). Mean KPS at last follow-up (see Table 2) was 70 (range 0–100). The difference was not statistically significant (EA mean 80, range 0–100; EI 70, range 0–100; *p* = 0.29).

### 3.3. Outcome

The mean follow-up was 1049 days (ranging from 13 to 5869 days).

Mean local recurrence-free survival (LRFS) was 697 days. A total of five patients (19.2%) suffered from a recurrence during this time. LRFS was longer in the EA group, although the difference did not reach statistical significance (EI 422 days, EA 1071 days, *p* = 0.06). LRFS was longer with adjuvant charged-particle therapy (744 days, vs. 296 days, *p* = 0.14).

Mean overall survival (OS) was 1049 days, again favoring the EA resection group (EI 910 days, EA 1238 days, *p* = 0.56); three patients died during the follow-up period, one patient at the age of 81 under unknown circumstances (unclear whether tumor related; underwent EA resection), one at the age of 68 (tumor progression with multiple metastases; underwent EI resection), and one at the age of 16 (due to uncontrollable bleeding from a metastasis in the lung; underwent EI resection).

**Figure 2:**
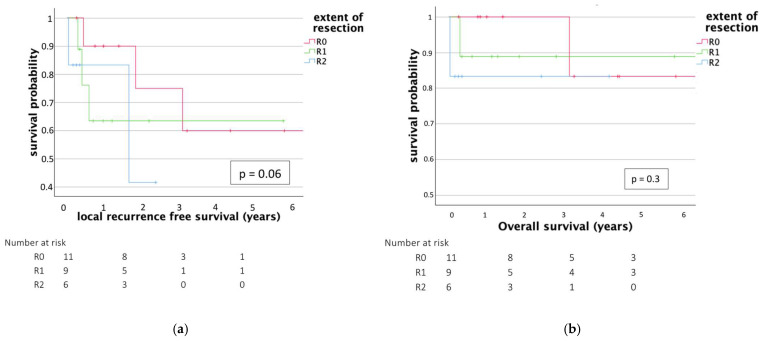
Kaplan–Meier curve of LRFS and OS after primary surgical treatment of mobile spine chordoma. Patients that underwent EA resection are depicted in red, patients that underwent EI resection without macroscopic tumor remnants are depicted in green and those with tumor remnants are depicted in blue. (**a**) no significant differences in LRFS (*p* = 0.06) or (**b**) OS (*p* = 0.3) could be shown.

Due to small sample size, a significant correlation between the extent of resection (R0 vs. R1 vs. R2) and OS or LPFS was not observed (Figure 2) (*p* = 0.3 (OS), *p* = 0.06 (LPFS)). Mean OS was longer with adjuvant radiotherapy (1375 days vs. 430 days, *p* = 0.099).

The two patients who had undergone emergency surgeries due to severe neurological deficits benefited from the decompression (patient IDs 13 and 8, see Table 2). Both were discussed in our interdisciplinary tumor board and subsequently underwent a second step with the removal of the residual tumor (EI group). Both were treated with adjuvant radiotherapy. One patient stayed without tumor recurrence during the follow-up period. The second patient suffered from a local recurrence after 607 days.

Complications during follow-up were investigated as well. Seven patients (27%) suffered from hardware failure: one screw breakage, one rod breakage, two cases of screw loosening, and three cases of cage dislocation. All of these cases required revision surgery, and none correlated to the surgical technique chosen (*p* = 0.29). No significant correlation was detected between hardware failure and other parameters, such as sex, age, tumor size, or extent of resection (see Table 3). Other factors like postoperative radiotherapy (OR, odds ratio = 1.5, 95% CI, confidence interval = 0.03–67.42, *p* = 0.83), EA resection (OR = 2.32, 95% CI = 0.36–15.06, *p* = 0.38), and the use of carbon fiber-only hardware (OR = 4.26, 95% CI = 0.31–59.11, *p* = 0.28) showed trends but also did not show any significant correlations

Table 3 is a logistic regression analysis assessing the impact of various risk factors on surgery-related complications in the follow-up period. The table presents the OR and hazard ratios (HR) with corresponding 95% CI and *p*-values for each factor, including patient sex, age, SINS score, tumor size, postoperative radiotherapy, en bloc resection, use of carbon fiber-only hardware, surgery duration, and blood loss.

### 3.4. Case Descriptions

Case 1: Chordoma of the lumbar spine, treated with en bloc resection

**Figure 3:**
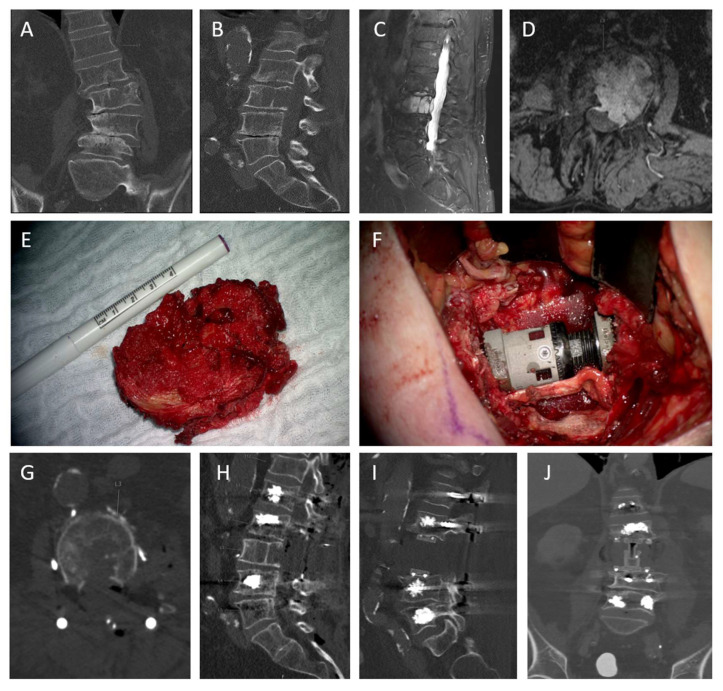
Pre- and postoperative imaging of a case of a chordoma of L3. (**A**,**B**): preoperative coronal and sagittal CT showing osteolysis and thoracolumbar scoliosis (**C**,**D**): sagittal and axial T1 contrast-enhanced MRI imaging showing the extent of the tumor (**E**): intraoperative photograph showing en bloc removed vertebra (**F**): intraoperative photograph showing carbon PEEK implant (**G**,**H**): CT axial and sagittal after first surgery (dorsal instrumentation, cementaugmentation, dorsal release) (**I**,**J**): sagittal and coronal CT after second step showing carbon implant according to [15].

The patient was a 75-year-old male with lower back pain for 6 months. MRI showed a tumor in L3, compromising all three vertebral columns (Figure 3). After discussion in our interdisciplinary tumor board, the decision for en bloc resection was made. Accounting for the differential diagnosis of a hemangioma, a preoperative angiography and embolization were performed. Afterwards, the surgery was conducted as follows: dorsal stabilization of L1–L5 with titanium screws (Solera, Medtronic, Dublin, Ireland) and cement augmentation as well as dorsal release as described in the methods section. Some days later, a corporectomy L3 and implantation of a distractible carbon cage and a piece of rib were conducted. Final postoperative imaging showed complete resection (R0). Postoperatively, the patient was pain-free, and radiotherapy was applied after six weeks.

Case 2: Chordoma C2 with acute neurological deficits

**Figure 4:**
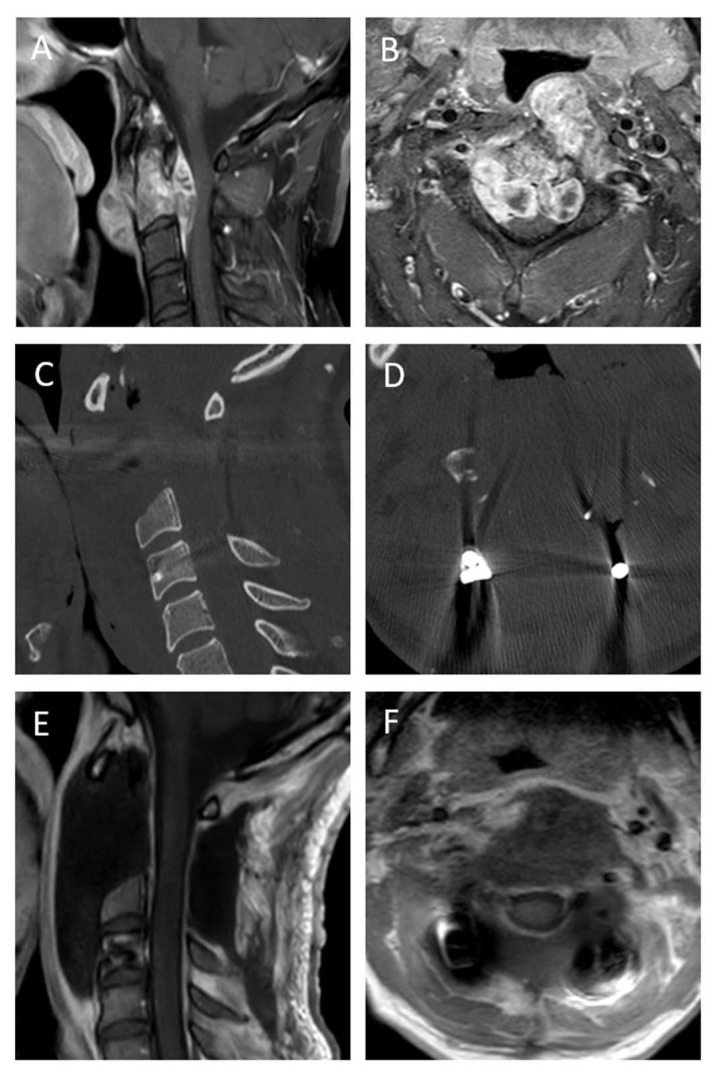
Pre- and postoperative imaging of a case of a chordoma of C2 with significant spinal cord compression. (**A**,**B**): Sagittal and axial T1 contrast-enhanced MRI imaging showing the extent of the tumor with ventral compression of the spinal cord, (**C**,**D**): Postoperative CT imaging after dorsal instrumentation of C1–4 with titanium lateral mass and pedicle screws and dorsal release and consecutive EI resection, (**E**,**F**): Postoperative T1 + contrast MRI imaging showing complete resection of the tumor.

The patient was a 42-year-old male with neck pain and a proximal paresis of his arms and ataxia. MRI imaging showed a large tumor at C2 with spinal cord compression (Figure 4). Considering the neurology, surgery had to be conducted within 24 h. Surgical treatment was conducted as follows: dorsal stabilization C1–4 with titanium screws and rods, laminectomy C2 and C3 and tumor debulking was performed. After histopathological diagnosis of chordoma and discussion in our interdisciplinary tumor board, resection of C2 and partially of C3 was conducted. Final postoperative MRI showed complete resection (R1). Postoperatively, the patient improved significantly, and the neurological deficits vanished completely. Radiotherapy was applied after 2 weeks.

## 4. Discussion

For many years, the standard of care for spinal chordomas consisted of maximal surgical resection, followed by adjuvant radiation or particle therapy [4]. This approach has been associated with the most favorable outcomes in terms of local recurrence-free survival rates (LRFSRs) and OS, both in newly diagnosed cases and in recurrences, as demonstrated by numerous studies [4,16,17,18,19,20].

However, chordomas of the mobile spine frequently preclude Enneking-appropriate resections with wide surgical margins due to anatomical constraints [21,22]. This challenge, along with two additional factors, has led to an increasing exploration of alternative treatment strategies and a growing acceptance of Enneking-inappropriate resections as a valid therapeutic option in recent years [7,17].

First, advances in particle therapy and accumulating evidence supporting its ability to improve LRFSRs and OS with acceptable toxicity profiles have shifted the treatment paradigm [23,24,25,26]. Second, recurrent reports of the inherent morbidity and mortality associated with en bloc resection—citing revision rates of up to 45% [27]—have further fueled this reconsideration.

As expected, the present study shows overrepresentation of EI approaches, as well as the only macroscopically positive margins in cervical lesions. Concerning chordomas of C2, an EA resection is nearly impossible. Molina et al. reviewed seven cases of chordomas of C1 and C2 and confirmed higher complication rates and less favorable resection margins than for chordomas of the subaxial spine [28]. In these cases, an EI approach or even a partial resection combined with radiotherapy seems like the best alternative. Recently published data suggests that mobile spine chordomas in general are significantly related to worse LRFS compared to sacral tumors, also pointing out the substantial challenges associated with their surgical therapy [29].

Both in the present study and in our previous reports [8], it has been demonstrated that en bloc resections can be performed with low complication rates and excellent long-term outcomes when carried out in specialized spine centers. In this series, the procedures were conducted using a minimally invasive, two-staged approach. The identification of a multistage surgical approach as an independent risk factor [5] for inferior outcomes in other studies may, in this case, be attributable to limited experience and insufficient routine with this surgical technique.

Despite the significantly higher intraoperative blood loss, the en bloc resection group did not exhibit a longer hospital stay, nor did it show a higher rate of short- or long-term complications. Clinical outcomes were very favorable and even slightly superior to those observed in the intralesional resection group. The time to RTX was especially low, at 28 days (EA) and 40 days (EI), respectively. Other studies show periods three times as long [5], which might be a key factor in the explanation of the EI group’s favorable survival results. We show a mechanical complication rate of 27%, which is comparatively low and particularly not higher than in the EI group [5,27].

A similar case series of De Robertis et al. reviewed 33 patients with mobile spine chordomas and showed no significant differences in OS and LRFS between EI and EA resection groups [5]. They achieved R0 resections in 36% of the cohort (in line with 42% in this study) and showed a LRFS of 36 months without significant differences between the groups. In our study cohort, LRFS was substantially longer with EA resection (EA median 35 months vs. EI resection median 14 months), whereas adjuvant charged-particle therapy prolonged LRFS independently of surgical resection. We also showed a substantial impact of radiotherapy on OS (46 months vs. 14 months, *p* = 0.099).

Although statistical significance could not be reached due to small sample sizes, emerging trends towards intralesional resections followed by adjuvant therapies cannot be supported through this data. Emphasis should continue to be placed on achieving the most complete surgical resection possible, followed by adjuvant radiotherapy.

## 5. Limitations

The present study has a few limitations, primarily due to the small sample size, which reflects the rarity of chordomas and the restricted indications for vertebrectomy. Other limitations were the unicentric nature of this study, the heterogeneity of the surgical outcomes and follow-up data, and the fact that this study was conducted retrospectively. Prospective, multicenter studies are recommended to further investigate this topic.

## 6. Conclusions

En bloc resection remains the gold standard for mobile spine chordoma resection whenever feasible. Nevertheless, Enneking-appropriate resection is often limited by anatomical constraints. In such cases, every effort should still be made to achieve the most complete tumor resection possible prior to initiating adjuvant therapy.

## Figures and Tables

**Table 1:** Comparison of EA and EI groups.

Characteristic	Enneking-Appropriate Resection EA	Enneking-Inappropriate Resection EI	*p*-Value
Number of patients	11	15	
Sex (male)	8 (72.7%)	11 (73.3%)	0.87
Age (mean, range)	64 (41–82)	51 (16–88)	0.81
CCI (mean, range)	4.63 (2–7)	5.07 (2–19)	0.71
BMI (mean, range)	28.41 (22–45.2)	26.97 (22.2–37)	0.93
Tumor size (mean, range)	43.4 mm (20–94)	38.9 mm (18–83)	0.53
Previous RTX	4 (36.2%)	4 (26.6%)	0.61
LOH (days) (mean, range)	20 (8–57)	21 (5–60)	0.9
Blood loss (mean)	4690 mL	1775 mL	0.027
Surgery duration (mean)	467 min	312 min	0.078
Postoperative radiotherapy	8 (72.7%)	9 (60%)	0.62
Time to radiotherapy (days, mean)	28	40	0.49
LRFS (days) (mean, range)	1071 (96–3670)	422 (13–2119)	0.06
OS (days) (mean, range)	1238 (96–4460)	910 (13–5869)	0.56

CCI: Charlson Comorbidity Index, BMI: Body Mass Index, RTX: Radiotherapy, LOH: Length of Hospitalization, LRFS: Local Recurrence-Free Survival, OS: Overall Survival.

**Table 2:** Case summary of all 26 patients showing their therapy, outcome and follow-up.

	Patient Characteristics									Surgery					Follow Up							
**Pat. ID**	Age	Sex	Localisation	CCI	BMI	KPS	SINS Score	Enneking	Previous Therapies	Date of Surgery	Resection Margins	Surgery Time min	Blood Loss mL	Number of Surgeries	LOH Days	Short-Term Complications	Length Off Follow Up Days	Long Term Complications	Adjuvant Radio- Therapy	KPS At Last Follow Up	Death	Recurrent Tumor
1	26	male	cervical	2	24.1	90	8	1B	surgery, radiotherapy	6 June 2008	R1	172	900	1	5	-	220	-	0	100	-	1
2	45	male	cervical	2	22.2	90	7	1B	-	13 May 2009	R1	73	650	2	7	-	5869	-	1	100	-	-
3	25	male	cervical	2	27.8	60	8	1B	-	28 November 2011	R2	175	700	1	24	-	92	-	0	70	-	-
4	62	male	lumbar	4	25.5	90	11	1B	-	22 March 2013	R0	148	500	2	8	-	4460	-	1	100	-	-
5	64	male	thoracic	4	37.9	50	13	2B	-	1 February 2014	R0	608	3200	2	57	-	1133	hardware failure	1	0	+	-
6	44	female	cervical	5	34.4	90	8	1A	surgery	17 March 2014	R2	102	600	2	21	-	126	-	1	100	-	-
7	55	female	lumbar	3	22.7	80	7	2B	radiotherapy	30 November 2015	R0	224	4000	4	17	-	1584	-	1	90	-	1
8	65	male	cervical	4	26.4	80	11	3	surgery, radiotherapy	5 December 2016	R1	114	200	2	23	-	462	-	0	80	-	1
9	73	male	lumbar	6	23.7	80	11	2A	-	24 May 2018	R0	446	10,000	2	30	-	295	-	0	70	-	1
10	64	male	thoracic	4	26.1	80	14	2B	surgery	26 March 2019	R1	524	3000	2	17	-	2119	-	1	40	-	-
11	41	male	lumbar	2	27.2	80	10	2B	neoadjuvant radiotherapy	16 July 2019	R0	549	1000	2	9	-	1598	hardware failure	0	80	-	-
12	71	female	cervical	5	27.7	80	6	2B	-	7 August 2019	R0	350	5000	3	10	-	2131	-	1	90	-	-
13	42	male	cervical	4	24.5	80	11	2B	-	30 October 2020	R2	339	5000	2	13	-	1504	-	1	90	-	1
14	81	female	thoracic	15	24.2	60	6	2B	-	29 July 2021	R1	277	800	3	24	wound	108	-	1	0	+	-
15	67	female	cervical	4	45.2	90	8	2B	-	17 March 2022	R0	86	200	2	9	-	1178	hardware failure	1	100	-	-
16	73	male	lumbar	6	30.9	80	8	3	surgery, radiotherapy	25 May 2022	R1	538	1900	2	11	-	124	hardware failure	0	80	-	-
17	43	male	cervical	4	31.1	40	9	3	surgery	17 June 2022	R2	243	3300	2	60	-	60	-	0	10	-	-
18	31	male	cervical	2	23.8	100	7	2A	neoadjuvant radiotherapy	1 September 2022	R1	450	1100	3	10	-	1010	hardware failure	0	100	-	-
19	56	female	cervical	4	25.7	90	7	2B	-	4 October 2022	R2	490	1900	2	22	pulm. Embolism	869	-	1	70	-	-
20	16	male	cervical	6	22.5	100	9	3	surgery, radiotherapy	4 July 2023	R2	250	180	2	13	-	13	-	0	0	+	-
21	66	male	lumbar	9	37.0	80	8	2B	surgery	14 August 2023	R1	377	3500	2	7	cage malposition	663		1	80	-	-
22	53	male	lumbar	3	26.8	90	11	2B	radiotherapy	15 January 2024	R0	585	3700	2	9	-	509	hardware failure	1	90	-	-
23	88	female	lumbar	7	23.9	100	9	3	-	26 April 2024	R1	557	2900	2	58	wound	407	-	1	100	-	-
24	62	male	cervical	7	22.0	80	10	2B	surgery, radiotherapy	14 June 2024	R0	826	15,000	2	36	meningitis	358	-	1	60	-	-
25	75	male	lumbar	6	28.4	80	8	2B	-	5 September 2024	R0	411	2000	2	26	-	275	-	1	90	-	-
26	82	male	lumbar	7	25.4	80	11	2B	-	3 March 2025	R0	912	7000	2	11	-	96	-	1	80	-	-

**Table 3:** Logistic regression analysis for surgery-related complications in the follow-up period.

Risk Factors	OR (95% CI)	*p*	HR (95% CI)	*p*
Sex (Male)	0.37 (0.01–23.66)	0.64	0.46 (0.03–22.78)	0.59
Age	1.01 (0.92–1.09)	0.87	1.02 (0.89–1.13)	0.76
SINS score	0.87 (0.43–1.74)	1.00	0.84 (0.56–1.17)	0.82
Tumor size	0.95 (0.85–1.05)	0.28	0.97 (0.88–1.07)	0.34
Postoperative radiotherapy	1.5 (0.03–67.42)	0.83	1.3 (0.08–52.34)	0.56
En bloc resection	2.32 (0.36–15.06)	0.38	1.9 (0.55–14.07)	0.34
Carbon fiber-only hardware	4.26 (0.31–59.11)	0.28	3.89 (0.47–49.22)	0.54
Surgery duration	1.01 (0.99–1.03)	0.09	1.24 (0.97–1.39)	0.37
Blood loss	0.99 (0.98–1.00)	0.18	0.97 (0.94–1.01)	0.29

## Data Availability

Data are contained within the article.
